# Supplementary material for: Signal voids of active cardiac implants at 3.0 T CMR
Source: Sci Rep. 2022 Apr 15;12:6285. doi: 10.1038/s41598-022-09690-z (PMC9014817; doi:10.1038/s41598-022-09690-z)
Supplement: Supplementary file 1 — Supplementary Information. [file 41598_2022_9690_MOESM1_ESM.pdf]

Supplemental File

Signal Voids of active cardiac implants at 3.0T CMR

Theresa Reiter[1,*], Ingo Weiss[2], Oliver M. Weber[3], Wolfgang R. Bauer[1]

[1] University Hospital Wuerzburg, Department of Internal Medicine I, Cardiology, Wuerzburg, Germany

[2] BIOTRONIK SE & Co. KG, Berlin, Germany

[3] Philips GmbH, Hamburg, Germany

* Corresponding author: Dr. Theresa Reiter, MD, University Hospital Wuerzburg, Department of Internal Medicine I, Cardiology, Oberduerbacher Strasse 6a, 97080 Wuerzburg, Germany. Reiter_t@ukw.de.

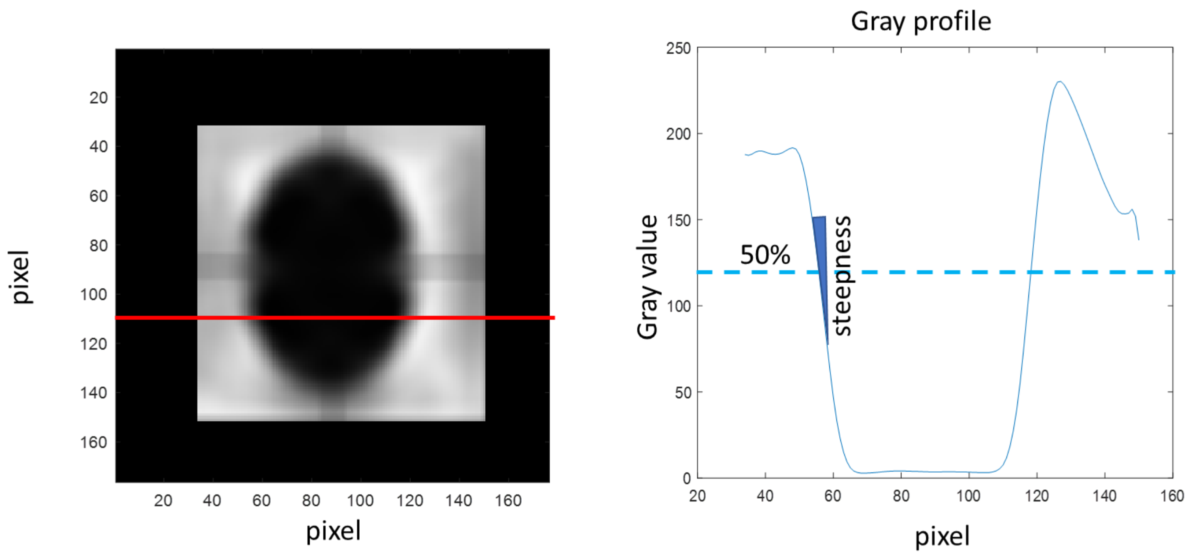

Supplemental figure 1: Example of a gray profile for a specific location. For the analysis of the signal void, the brightest signal within the whole volume data set was defined as 100%.

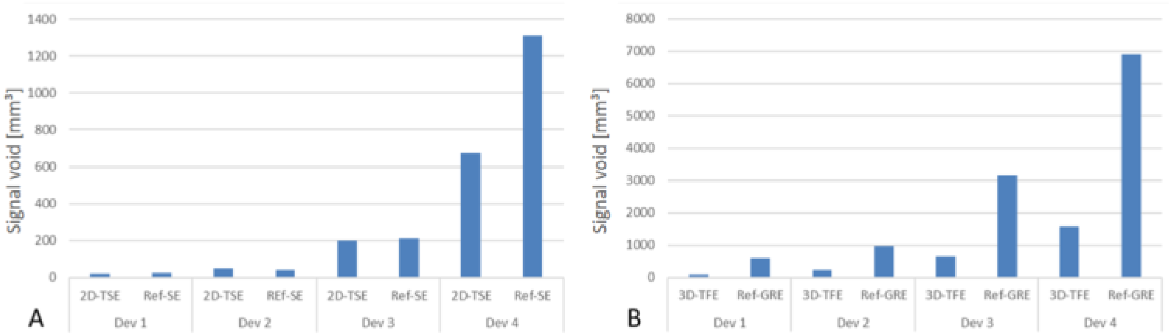

Supplemental figure 2: A) Comparison of the signal voids caused by the four devices when measured with the 2D-TSE sequence and the reference sequence. b) Comparison of the signal voids caused by the four devices when measured with the 3D-TFE sequence and the reference sequence. All measurements were performed with the orientation 1.

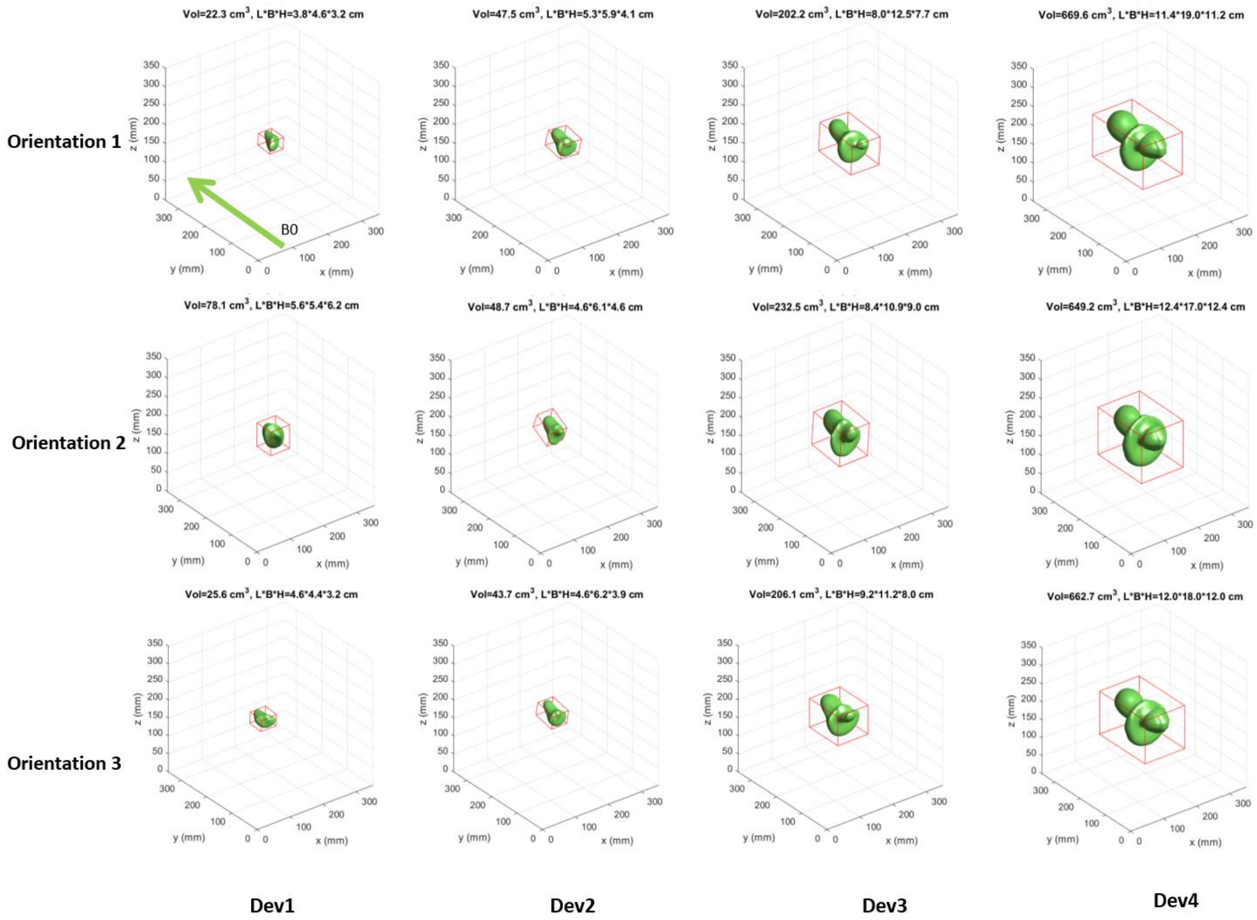

Supplemental Figure 3: 2D-TSE induced signal void volumes and bounding box for all four devices measured in three orientations.

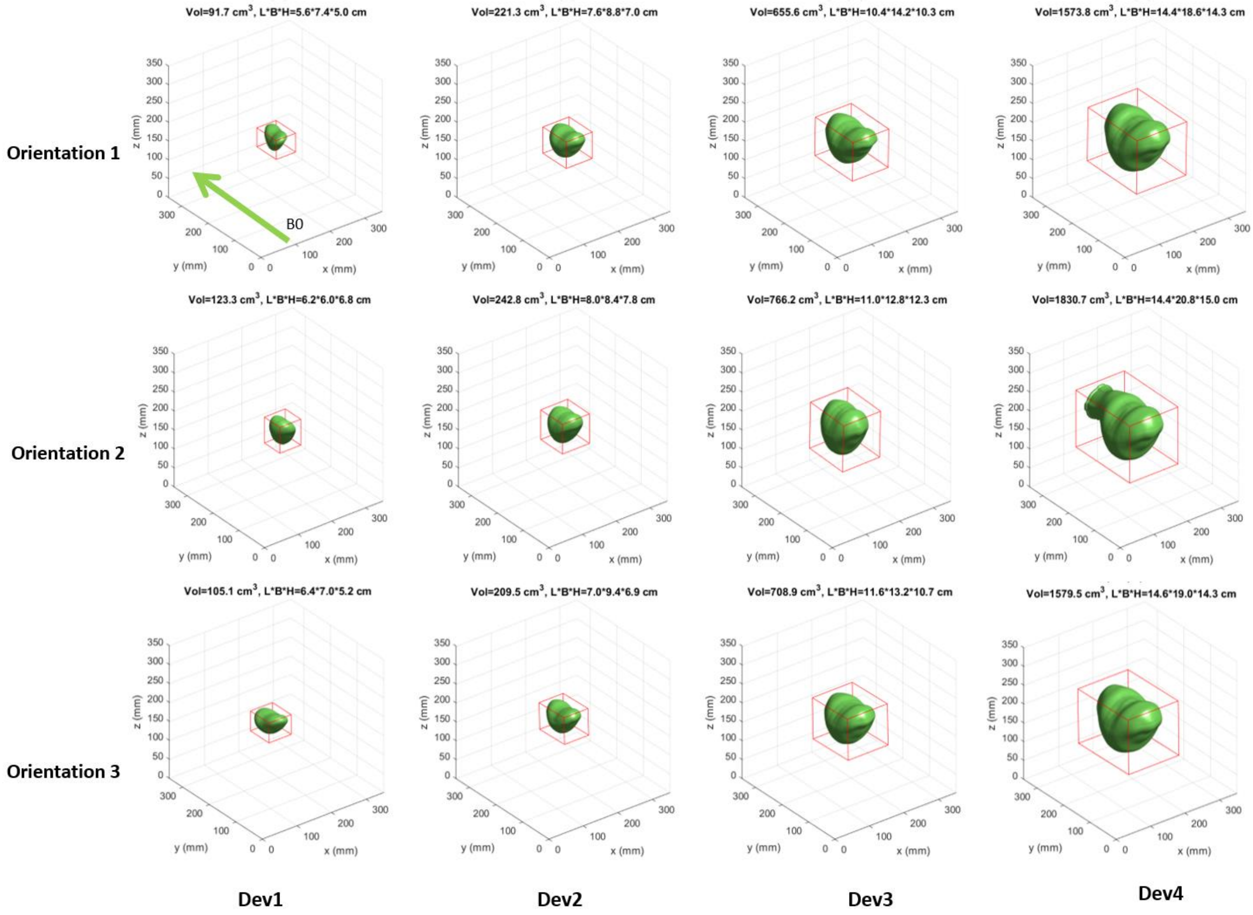

Supplemental Figure 4: 3D-TFE induced signal void volumes and bounding box for all four devices measured in three orientations.
